# Antimicrobial Resistance, Biofilm Formation, and Virulence Genes in *Enterococcus* Species from Small Backyard Chicken Flocks

**DOI:** 10.3390/antibiotics11030380

**Published:** 2022-03-13

**Authors:** Othman M. Alzahrani, Mahmoud Fayez, Amal S. Alswat, Mohamed Alkafafy, Samy F. Mahmoud, Theeb Al-Marri, Ahmed Almuslem, Hassan Ashfaq, Shaymaa Yusuf

**Affiliations:** 1 Department of Biology, College of Science, Taif University, P.O. Box 11099, Taif 21944, Saudi Arabia; o.alzahran@tu.edu.sa; 2 Al-Ahsa Veterinary Diagnostic Lab, Ministry of Environment, Water and Agriculture, Al-Ahsa 31982, Saudi Arabia; theep8@hotmail.com (T.A.-M.); aaa4481@hotmail.com (A.A.); 3 Department of Bacteriology, Veterinary Serum and Vaccine Research Institute, Ministry of Agriculture, Cairo 11381, Egypt; 4 Department of Biotechnology, College of Science, Taif University, P.O. Box 11099, Taif 21944, Saudi Arabia; a.alswat@tu.edu.sa (A.S.A.); m.kafafy@tu.edu.sa (M.A.); s.farouk@tu.edu.sa (S.F.M.); 5 Institute of Continuing Education and Extension, University of Veterinary Animal Sciences, Lahore 54000, Pakistan; hasan.ashfaq@uvas.edu.pk; 6 Department of Microbiology, Faculty of Veterinary Medicine, Assiut University, Assiut City 71515, Egypt

**Keywords:** antimicrobial resistance, *Enterococcus*, backyard chickens, virulence genes, multidrug resistance, antimicrobial-resistance genes

## Abstract

Backyard birds are small flocks that are more common in developing countries. They are used for poultry meat and egg production. However, they are also implicated in the maintenance and transmission of several zoonotic diseases, including multidrug-resistant bacteria. Enterococci are one of the most common zoonotic bacteria. They colonize numerous body sites and cause a wide range of serious nosocomial infections in humans. Therefore, the objective of the present study was to investigate the diversity in *Enterococcus* spp. in healthy birds and to determine the occurrence of multidrug resistance (MDR), multi-locus sequence types, and virulence genes and biofilm formation. From March 2019 to December 2020, cloacal swabs were collected from 15 healthy backyard broiler flocks. A total of 90 enterococci strains were recovered and classified according to the 16S *rRNA* sequence into *Enterococcus faecalis* (50%); *Enterococcus faecium* (33.33%), *Enterococcus hirae* (13.33%), and *Enterococcus avium* (3.33%). The isolates exhibited high resistance to tetracycline (55.6%), erythromycin (31.1%), and ampicillin (30%). However, all of the isolates were susceptible to linezolid. Multidrug resistance (MDR) was identified in 30 (33.3%) isolates. The enterococci AMR-associated genes *ermB*, *ermA*, *tetM*, *tetL*, *vanA*, *cat,* and *pbp5* were identified in 24 (26.6%), 11 (12.2%), 39 (43.3%), 34 (37.7%), 1 (1.1%), 4 (4.4%), and 23 (25.5%) isolates, respectively. Of the 90 enterococci, 21 (23.3%), 27 (30%), and 36 (40%) isolates showed the presence of *cylA*, *gelE,* and *agg* virulence-associated genes, respectively. Seventy-three (81.1%) isolates exhibited biofilm formation. A statistically significant correlation was obtained for biofilm formation versus the MAR index and MDR. Multi-locus sequence typing (MLST) identified eleven and eight different STs for *E. faecalis* and *E. faecium*, respectively. Seven different rep-family plasmid genes (rep1–2, rep3, rep5–6, rep9, and rep11) were detected in the MDR enterococci. Two-thirds (20/30; 66.6%) of the enterococci were positive for one or two rep-families. In conclusion, the results show that healthy backyard chickens could act as a reservoir for MDR and virulent *Enterococcus* spp. Thus, an effective antimicrobial stewardship program and further studies using a One Health approach are required to investigate the role of backyard chickens as vectors for AMR transmission to humans.

## 1. Introduction

Enterococci are Gram-positive bacteria that belong to the commensal microbiota of humans, animals, and poultry [1]. They are ubiquitous in nature and can be found in soils, freshwater, and plants [2]. *Enterococcus* spp. are important opportunistic human pathogens that are responsible for a wide range of serious nosocomial infections, including bacteremia, urinary tract infections, endocarditis, and intra-abdominal infections [3,4,5].

In veterinary medicine, enterococci are particularly significant as the causative agent of different infections, such as mastitis in cattle, bacteremia in dogs and pigs [6,7], and septicemia, endocarditis, amyloid arthropathy, and spondylitis in poultry [8,9].

Antibiotic resistance is an emerging world health threat. Many bacteria have developed resistance to frequently used antibiotics due to the unregulated use of antimicrobials in humans, agriculture, animals, poultry husbandry, and aquaculture in many developing countries [10,11].

One of the major concerns regarding opportunistic pathogens is their frequent antimicrobial-resistance (AMR) profile. Enterococci are intrinsically resistant to commonly used antibiotic classes, such as cephalosporins, β-lactams, sulfonamides, and are resistant at variable levels to aminoglycosides. Moreover, they are able to acquire resistance to clinically relevant drugs via horizontal transfer [12,13]. Enterococci are thought to play a key role in the acquisition, conservation, and transmission of AMR genes to other bacteria [14].

Enterococci pathogenesis is attributed to a variety of virulence factors. The most important adhesion factors that play a role in biofilm formation are *Asa* (aggregation substance), *Esp* (extracellular surface protein), *EfaA* (*E. faecalis* antigen A), *Ace* (adhesin of collagen from *E. faecalis*), and *Ebp* (endocarditis and biofilm-associated pili). Enterococci secrete the pathogenic factors *CylA* (cytolysin) and *GelE* (gelatinase), which are responsible for the exacerbation of infection. The expression of these factors is essential for biofilm formation, attachment, invasion, and the secretion of toxins [1,15,16,17]. 

Several molecular typing methods have been used to type enterococci, including pulsed-field gel electrophoresis, random amplification of polymorphic DNA, repetitive sequence-based PCR, ribotyping, and multi-locus sequence typing (MLST) [18,19,20,21,22,23]. MLST is a preferable tool for several pathogens, especially when epidemiological, geographical, and evolutionary studies need to be carried out [24,25]. Two MLST schemes have been developed for typing *E. faecalis* and *E. faecium* based on differences in the sequences of seven housekeeping genes (*gdh, gyd, pstS, gki, aroE, xpt,* and *yiqL*) for *E. faecalis* and (*adk, atpA, ddl, gyd, gdh, purK,* and *pstS*) for *E. faecium* [22,23].

One of the major public health concerns related to enterococci is their frequent antimicrobial resistance (AMR). Human infection by AMR enterococci occurs mainly by consuming contaminated meat or meat products from poultry and other livestock [26,27,28], and contamination can occur during slaughtering and evisceration [29].

Enterococci have a high capacity to acquire antimicrobial resistance either by point mutation or by the horizontal transfer of genetic elements [30,31,32]. Conjugation is thought to be the most common way of exchanging genetic elements, either by conjugative transposons or by horizontal transfer of plasmids [33].

In general, enterococcal plasmids are classified into two groups: a conjugative group consisting of pheromone-responsive and non-pheromone-responsive plasmids and a nonconjugative group consisting of small rolling-circle replicating (RCR) and mosaic plasmids [34].

Inc18 plasmids and pMG1-type plasmids are classified as conjugative non-pheromone-responsive plasmids, and frequently carry antibiotic resistance genes [34,35]. Nonconjugative RCR plasmids are typically small, with a high copy number, and a broad host range. Moreover, they frequently contain antibiotic resistance genes [36]. Jensen et al. [37] recently developed a classification scheme for enterococci based on the replication–initiation genes (rep) and distinguished 19 families and various unique rep genes.

Backyard birds are small flocks that are common in developing countries. They are also very popular in the USA, where birds are raised for meat production. Direct contact between poultry and humans is frequent; thus, these backyard flocks are considered a vehicle for disease transmission. Moreover, backyard poultry could be an emerging predisposing cause for MDR pathogenic bacteria, which can disseminate among humans [38]. In Saudi Arabia, many studies highlighted the prevalence of MDR enterococci in hospitals and communities [39,40]. The first vancomycin-resistant *Enterococcus* was detected in 1992 [41]; however, information concerning enterococci in backyard chicken is scarce.

Consequently, this study aimed to investigate the antimicrobial resistance, virulence determinants, and biofilm formation, and to characterize the plasmid content and multi-locus sequence types in *Enterococcus* isolates from healthy chickens in backyard farms to highlight their zoonotic importance.

## 2. Results

### 2.1. Bacterial Isolation and Identification

Ninety *Enterococcus* isolates were isolated from 15 backyard chicken flocks and were biochemically identified into four species. The predominant species were *Enterococcus faecalis* (*E. faecalis*) (50%), followed by *Enterococcus faecium* (*E. faecium*) (33.33%), *Enterococcus hirae* (*E. hirae*) (13.33%), and *Enterococcus avium* (*E. avium*) (3.33%). Genetically, the *16S rRNA* sequences showed more than a 99% homology with the relevant enterococci in the NCBI database. They were deposited in the NCBI sequence database with GenBank accession numbers OL691094-OL691103, OL677341-OL677350, OL691538-OL691543. On the basis of the 16S rRNA sequence analysis, 45 *E. faecalis*, 30 *E. faecium*, 12 *E. hirae,* and 3 *E. avium* isolates were clustered with the reference enterococci (*E. faecalis* NR_040789.1, *E. faecium* NR_042054.1, *E. hirae* NR_037082.1, and *E. avium* NR_028748.1), with a similarity level of 100% Supplementary Figure S1.

### 2.2. Antimicrobial Sensitivity

**Figure 1:**
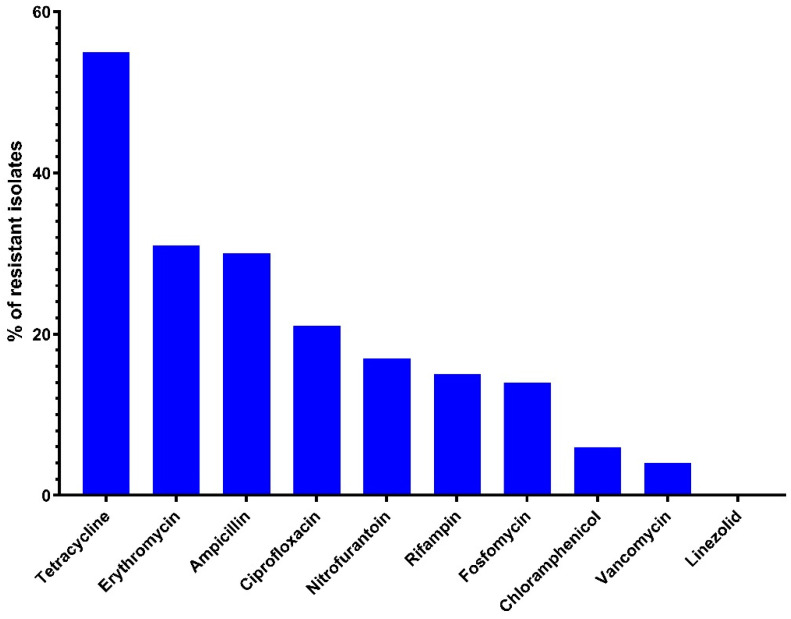
Frequency of antimicrobial resistance of Enterococci isolates (*n* = 90) recovered from healthy backyard chickens.

The antimicrobial-resistance profile of the 90 *Enterococcus* isolates is shown in Figure 1. Sixteen isolates (17.7%) were susceptible to all antibiotics. Resistance to one or more antimicrobials was determined in 74 (82.22%) enterococci. The MIC values and the resistance levels of 90 enterococci to 10 different antimicrobials are shown in Table 1 and Supplementary Table S1. Resistance to tetracycline (55.6%), erythromycin (31.1%), ampicillin (30%), ciprofloxacin (21.1%), and nitrofurantoin (17.8%) were the most frequent. Conversely, none of the isolates were resistant to linezolid, and four isolates (3.3%) showed resistance to vancomycin. *E. faecalis* showed a high frequency of resistance to tetracycline (62.2%), rifampin (24.4%), and nitrofurantoin (22.2%), while *E. faecium* exhibited a high resistance to tetracycline (50%), ampicillin (30.3%), and ciprofloxacin (23.3%). 

Of the 74 resistant enterococci, 9 isolates (10%) were resistant to one antimicrobial agent, 35 (38.9%) showed resistance to two antimicrobials, and the remaining 30 (33.3%) showed MDR. The MDR enterococci isolates were distributed into 15 *E. faecalis* (33.3%), 8 *E. faecium* (26.7%), and 5 *E. hirae* (41.6%) isolates. The mean MAR index was 0.22 for *E. faecalis* (range: 0.1 to 0.5), 0.3 for *E. avium*, 0.23 for *E. faecium* (range: from 0.1 to 0.4), and 0.25 for *E. hirae*. (range: from 0.1 to 0.4). Table 2 shows the resistance profile of the 90 enterococcus isolates.

### 2.3. Antimicrobial-Resistance Genes

Nine antimicrobial-resistance genes were detected among the enterococci. Of the vancomycin-resistant isolates, 33.3% (1/3) contained *vanA*, while *vanB* was not detected in the three isolates. Twenty-four (86%) phenotypically erythromycin-resistant isolates were positive for erythromycin-resistant genes; the *ermB* gene was detected in all isolates, whereas the *ermA* gene was only detected in 11 isolates. Four tetracycline resistance genes (*tetM, tetA*, *tetB*, and *tetL*) were found in 94% (47/50) of tetracycline-resistant isolates. The prevalent resistance genes were *tetM* and *tetL*, accounting for 78% and 68% of the isolates, respectively; however, *tetA* and *tetB* were detected in 2% of the isolates. The *cat* gene was detected in 66% of the chloramphenicol-resistance isolates and the *pbp5* gene for ampicillin resistance was detected in 22 enterococci isolates. Table 3 shows the distribution of antimicrobial-resistance genes among different enterococci.

### 2.4. Biofilm Formation

Overall, 73 *Enterococcus* isolates (81.1%) were biofilm producers, among which 39 were *E. faecalis*, 24 were *E. faecium*, 8 were *E. hirae*, and 2 were *E. avium*. The isolates were further classified into four categories based on the OD of the bacterial biofilm: 17 non-biofilm producers (18.9%), 16 weak biofilm producers (17.8%), 29 medium biofilm producers (32.2%), and 28 strong biofilm producers (31.1%). *E. faecalis* showed a significantly higher biofilm formation (*p* < 0.0001), and 13 *E. faecalis* isolates (28.9%) exhibited strong biofilm formation.

**Figure 2:**
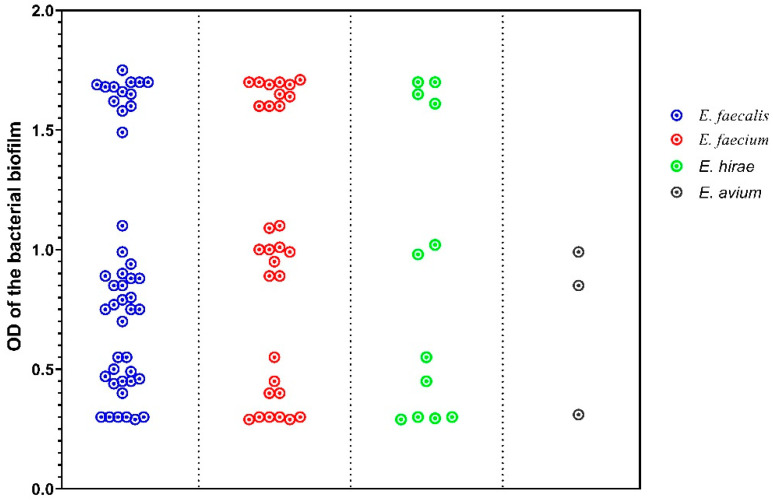
OD_570_ values indicate the amounts of bacterial biofilm among the Enterococcus species (*n* = 90): non-biofilm producers (0.29–0.31); weak producers (0.4–0.55); medium producers (0.7–1.1); and strong producers (1.4–1.7).

Figure 2 shows the biofilm formation strength of the *Enterococcus* species. A statistically significant pairwise correlation (*p* < 0.001) was obtained for biofilm formation versus MAR index (r = 0.807) and MDR (r = 0.639). Table 4 shows the bacterial biofilm OD MAR index mean values in the four categories. 

### 2.5. Gelatinase and Cytolysin Activity

**Figure 3:**
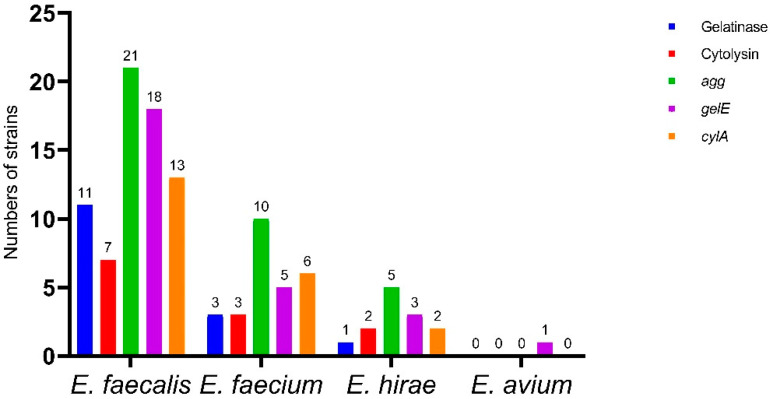
Distribution of *gelE* (gelatinase), agg (aggregation substance), and *cylA* (activator of cytolysin) genes, and gelatinase and cytolysin activity in the 90 *Enterococcus* isolates.

**Figure 4:**
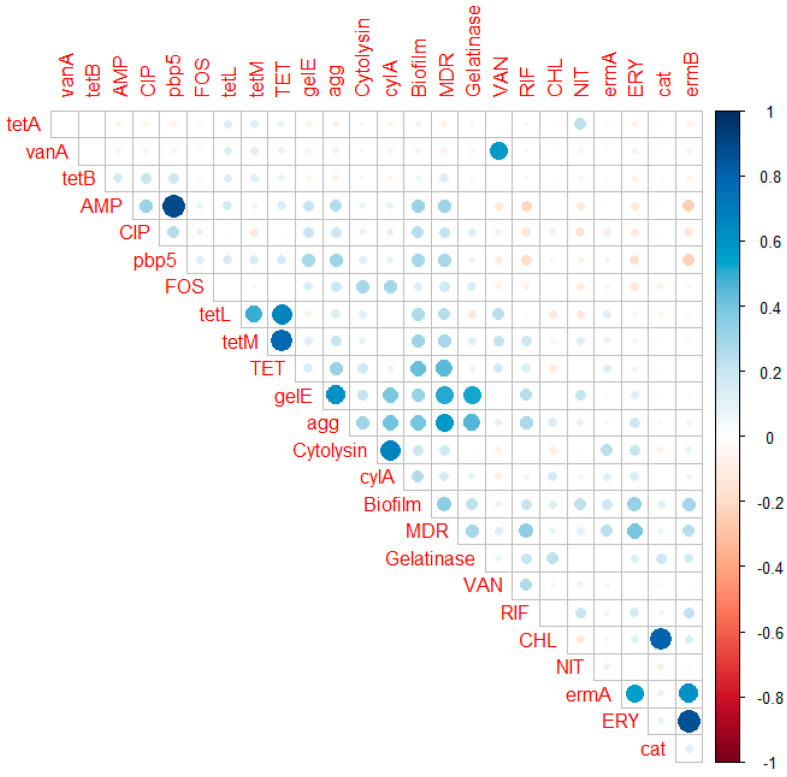
Correlation matrix of phenotypic (biofilm formation ability, gelatinase activity, hemolytic activity, and antibiotic resistance) and genotypic (*agg*, *gelE*, *cylA*, and antibiotic-resistance genes) features exhibiting a significant (*p* < 0.05) correlation. White spaces are not significantly correlated. Significant positive correlation is represented by blue circles, whereas significant negative correlation is represented by red circles. The numerical value of the Phi correlation coefficient is represented by the size and strength of the color.

Fifteen *Enterococcus* isolates (16.7%) were gelatinase producing, and 12 exhibited cytolysin activity (13.3%) (Figure 3). *E. faecalis* showed significantly higher gelatinase and cytolysin activities (*p* < 0.0001). A statistically significant pairwise correlation (*p* < 0.001) was found between biofilm formation versus gelatinase activity (r = 0.245) and cytolysin activity (r = 0.386) (Figure 4).

### 2.6. Virulence Genes

Figure 3 shows the distribution of virulence genes in all of the isolates and can be summarized as follows: *agg* in 21 *E. faecalis* (46.7%), 10 *E. faecium* (33.33%), and 5 *E. hirae* (41.66%); *gelE* in 18 *E. faecalis* (40%), 5 *E. faecium* (16.7%), 3 *E. hirae* (25%), and 1 *E. avium* (33.3%); *cylA* in 13 *E. faecalis* (28.9%), 6 *E. faecium* (20%), and 2 *E. hirae* (16.6%). The virulence genes were significantly higher in *E. faecalis* isolates (*p* < 0.0001). Simultaneously, nine *E. faecalis* (20%), two *E. faecium* (6.7%), and one *E. hirae* (8.3%) were positive for the three tested genes. The distribution of virulence genes among different biofilm categories is shown in Table 4. A significant correlation (*p* < 0.0001) was found between *gelE* and gelatinase (r = 0.521), *cylA* and cytolysin (r = 0.7), *agg* versus MDR (r = 0.0577), and *gelE* versus MDR (r = 0.514) (Figure 4).

### 2.7. MLST of E. faecalis and E. faecium

**Figure 5:**
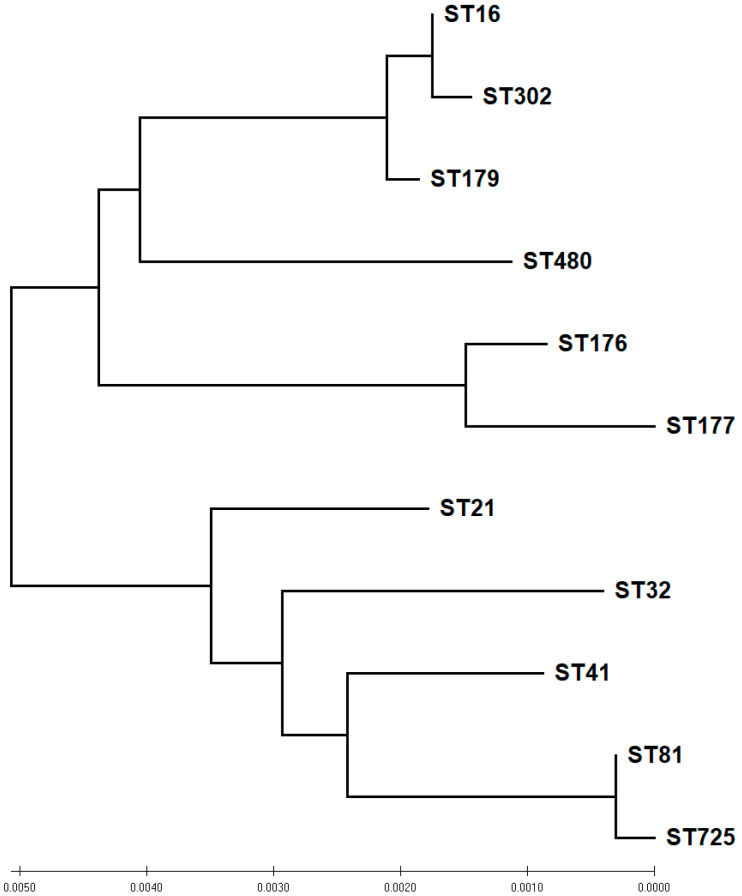
Phylogeny of sequence types (ST) of *E. faecalis* isolated from backyard chickens based on neighbor-joining analysis of concatenated sequences of the seven housekeeping genes used for multi-locus sequence typing (MLST).

MLST allelic profiles for *E. faecalis* and *E. faecium* are presented in Table 5 and Table 6. A total of 11 STs were found among the *E. faecalis* isolates. The most prevalent STs were ST16 (*n* = 10) and ST302 (*n* = 8), followed by ST179 (*n* = 6), ST480 (*n* = 5), and ST752 (*n* = 3) (Table 5). On the basis of the eBurst analysis and the phylogenetic analysis of the concatenated MLST sequences, seven STs were clustered into three groups: the first contained ST16, ST179, and ST302; the second ST81 and ST725; and the third ST176 and ST177; the remaining four STs (ST21, ST32, ST41, and ST480) were identified as singletons (Figure 5). 

**Figure 6:**
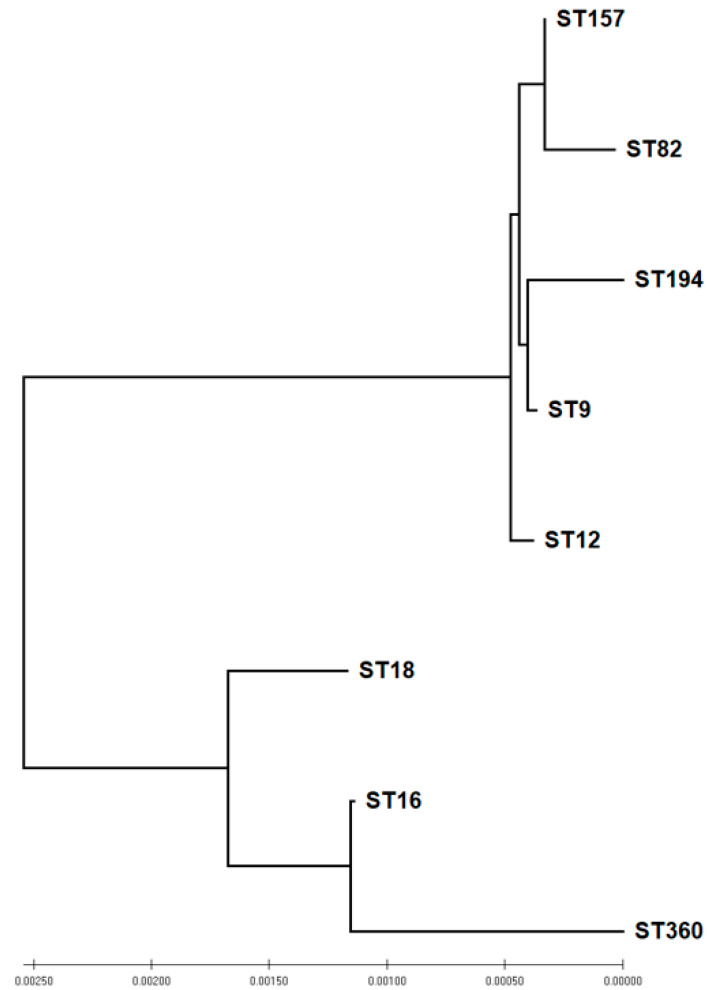
Phylogeny of sequence types (ST) of *E. faecium* isolated from backyard chickens based on neighbor-joining analysis of concatenated sequences of the seven housekeeping genes used for multi-locus sequence typing (MLST).

Eight STs were identified among the *E. faecium* isolates. The most abundant ST was ST194 (*n* = 8), followed by ST157 (*n* = 5), ST82 (*n* = 5), and ST9 (*n* = 4) (Table 6). The eBurst analysis shows that the registered isolates belong to five major clonal complexes (CC) in the order of their size: CC17, CC9, CC22, CC5, and CC94. Accordingly, ST9, ST157, ST82, ST194, and ST12 identified in this work are part of CC9, and ST16, ST18, and ST360 are part of the globally dispersed clonal lineage CC17 (Figure 6).

### 2.8. repA Genes (Plasmid Families)

Seven different rep-family plasmid genes (rep1–2, rep3, rep5–6, rep9, and rep11) were detected in the MDR enterococci. Two-thirds (20/30; 66.6%) of the enterococci were positive for one or two rep-families (Table 7). Seven out of fifteen *E. faecalis* (46.6%), two out of eight *E. faecium* (25%), one out of five E. hirae (20%), and one E. avium did not yield an amplicon for the rep-families. The most prevalent rep-family among *E. faecalis* was rep9 (pCF10), which was found in six isolates, followed by rep6 (pS86), which was found in three isolates; rep1 (pIP501) was found in one isolate. The predominant rep-family among *E. faecium* isolates was rep2 (pEF1071) with five isolates. Positive amplicons for rep6 (pS86) and rep1 (pIP501) were found in two and one isolates, respectively. Four different rep-families were found among *E. hirae*: rep5 (pN315) in three isolates, and each of rep3 (pAW63), rep6 (pS86), and rep11 (pEF1071) in one isolate. Positive amplicons for rep6 (pS86) and rep9 (pCF10) were found in the two *E. avium* isolates.

## 3. Discussion

Backyard chickens are considered to be a vector for disseminating several zoonotic diseases, including *Salmonella*, *Campylobacter*, enteropathogenic *E. coli*, and several antibiotic-resistant microorganisms [42,43,44]. Moreover, enterococci, notably *E. faecium* and *E. faecalis*, have emerged as major multidrug-resistant zoonotic bacteria due to the widespread use of antibiotics in human and veterinary treatments [45]. 

In the current study, four *Enterococcus* species were isolated from healthy backyard chickens. *E. faecalis* was the most predominant species, which is consistent with the results of previous studies [43,46,47]. In contrast, *E. faecium* was reported as the prevalent species in poultry [48,49]. 

Antimicrobial resistance is one of the characteristics of enterococci, and their ability to acquire and spread antibiotic resistance presents a challenge for infection control [50].

In this study, a high proportion of resistance was identified in the isolated enterococci, the majority showing tetracycline resistance, which is accordance with various studies [43,51,52,53]. Tetracycline-resistance genes (*tetM* and *tetL*) were detected in 78% and 68% of the isolates, respectively, while *tetA* and *tetB* genes were detected in 2% of the isolates. Tetracycline resistance is most often mediated by tetM and tetL in enterococci from humans, animals, food, and the environment [46,54,55,56,57]. Different tetracycline-resistance genes were identified in the *Enterococcus* species [28,58]. Phenotypic resistance to erythromycin and ampicillin was observed in 31.1% and 30% of isolates. The *ermB* gene was detected in 86% of isolates, whereas the *ermA* gene was detected in 11 isolates, which is in accordance with Mlynarczyk et al. [59] who described erm(B) as the most prevalent gene conferring erythromycin resistance in enterococci. The *pbp5* gene of ampicillin resistance was identified in 22 isolates, concordant with [43]. A high level of *Enterococcus* resistance to both tetracycline and erythromycin was reported in Switzerland [60], the Netherlands [61], France [62], and Portugal [63]. Macrolides, tetracyclines, and penicillins are the major antimicrobials used in integrated broiler companies [64], while in Saudi Arabia, tetracycline and erythromycin are the most frequently used antimicrobials in poultry farms [65]. Furthermore, tetracycline resistance has been described to co-select for erythromycin resistance [53]. The WHO classified macrolides as critically important (the highest priority) and tetracycline as highly important antimicrobials for human medicine [66].

Resistance to vancomycin has generated substantial research interest during the last decade since it is the drug of last resort to treat enterococci infections in humans [67]. In this work, we observed low vancomycin resistance (3.3%) among the isolated enterococci. However, the *vanA* gene was identified in all phenotypic-resistance isolates.

Our values are lower than those reported in other studies [68,69] and higher than the results of da Costa et al. [70]. However, Semedo-Lemsaddek et al. [43] did not detect any vancomycin resistance among their isolates. In the last decade, linezolid-resistant enterococci were detected in the USA, Europe, and Asia [50,71,72]. Remarkably, linezolid-resistant enterococci were not detected in our study. 

MDR was frequently detected among the isolated enterococci in this study. The emergence of MDR enterococci has also been reported worldwide, particularly in Korea (26.9%), Spain (87.5%), and Ethiopia (78.2%), and is currently regarded as a growing public health concern [72,73,74].

Biofilm formation plays a considerable role in enterococcal infections and antibiotic resistance [75,76]. In this study, biofilm formation was observed in 81.1% of the isolates, which is concordant with [75,76]. A positive pairwise correlation was observed between biofilm production and both the MAR index and MDR. Moreover, biofilm production has been linked to antibiotic resistance in enterococci [77]. In addition, the *gelE* gene was found in all biofilm-producing isolates but not in non-biofilm-producing isolates, suggesting its significance in biofilm development. *gelE* is necessary for biofilm formation because it stimulates cell aggregation in microcolonies, allowing them to construct a three-dimensional structure [78].

Despite *gelE* gene detection in 28% of isolates, in vitro gelatinase activity was only detected in 17% of isolates. Similarly, other investigations found that 30%, 56%, and 59% of clinical isolates generated gelatinase, while 90%, 88%, and 92% were *gelE* positive, respectively [79,80,81]. Together with our findings, these reports show that while *gelE* regulated by the *fsr* locus is necessary for gelatinase activity, it is insufficient since *fsrA* and *fsrB* are also required for the gelatinase phenotype.

Enterococcal cytolysin is a hemolytic virulence factor linked to human disease and increased patient mortality [82]. The *cylA* gene was detected in 21 isolates (23%), concordant with other studies in poultry [64,65]. However, only 57.1% of the isolates expressed hemolysin activity, which is in agreement with the results in [83,84,85]. These findings may be attributable to environmental factors such as the in vitro and in vivo conditions used to test for phenotypic characteristics, as these could have a significant impact on gene expression [86].

MLST represents an outstanding tool for global and long-term epidemiological studies. In this work, all 45 *E. faecalis* were divided into 11 STs. The most common STs in the backyard chickens (ST16, ST302, and ST179) have been previously found in poultry, wild birds, and pigs [63,87,88]. Furthermore, ST16, ST21, ST179, and ST480 were reported among *E. faecalis* hospital isolates in Saudi Arabia [89]. ST16 isolates were previously reported to display major diversity as to the source of isolation and lesions [22]. Among the ST16 *E. faecalis* isolates, two isolates were resistant to vancomycin. vanA *E. faecalis* ST116 isolates were previously isolated from turkey meat and non-hospitalized humans [67,90].

MLST genotyping of *E. faecium* isolates revealed eight different ST types, of which five belonged to CC9 and three belonged to CC17, suggesting an evolutionary link between backyard *E. faecium* isolates [91]. ST9, ST157, ST194, and CC9 in particular were previously isolated from poultry and poultry meat [92,93]. Although CC17 was reported as a nosocomial clonal complex [94], several studies reported the circulation of E. faecium CC17 in animals [67,93,95]. Backyard chickens possibly acquired the CC17 *E. faecium* isolates from contaminated environments, or humans visiting the farm. This suggestion is supported by a previous study that demonstrated the transmission of *E. faecium* of human origin to chickens [96]. Moreover, human-linked *E. faecium* has been isolated from various water and food sources [97,98].

Plasmids are believed to be plastic structures that change as a result of the ever-changing environment in which they reside [33]. In this work, a recently published scheme for plasmid classification was utilized in order to investigate whether specific plasmid families were involved in AMR in enterococcus from backyard chickens. Ten enterococci did not carry any plasmid of the rep-families. This result is concordant with the studies of Jensen et al. and Cho et al. [37,99], in which approximately one-third of their isolates sets from humans, animals, and environments did not yield any amplicons. However, a lower percentage of negative rep-families was reported elsewhere [100,101], where 4% and 1.3% of *E. faecium* and *E. faecalis* did not yield any amplicons, respectively.

Seven different rep-family plasmid genes were identified in this study, a value concordant with several previous studies that detected five to nine rep-family plasmid genes [37,54,102,103,104] and lower than the study of Cho et al. [99], who identified 12 rep-family plasmid genes.

In this study, rep9 (pCF10) and rep2 (pEF1071) were the predominant plasmid among *E. faecalis* and *E. faecium,* respectively. These two rep-families were also reported in earlier studies [37,54,102,103,104]. The linkage between both Inc18 plasmids, mainly represented by rep2pRE25, and pheromone-responsive plasmids, represented by rep9 pCF10 and both tetracycline and glycopeptide, was previously reported [105,106,107].

Studies on E. hirae revealed the detection of rep5 (pN315), rep3 (pAW63), and rep11 (pEF1071). These rep-families had not been previously detected among *E. faecalis* and *E. faecium* isolates [37,54,102,103,104]. However, Cho et al. [99] reported these rep-families among *E. hirae*, *E. casseliflavus*, *E. gallinarum*, and *E. mundti*. This finding may suggest diverse plasmid contents among different species of Enterococcus.

A limitation of this study was the lack of environmental samples from the investigated flocks. However, in a previous study [108], a diversity of *Enterococcus* species was isolated from farm environments with multiple antibiotic-resistance profiles, indicating the role of chicken in environmental contamination. Furthermore, this was a small study that could not include additional samples of *Enterococcus* species taken from this region. 

## 4. Materials and Methods

### 4.1. Study Area

The study was conducted in Al-Ahsa Governorate, in the eastern region of Saudi Arabia (25°22′44.1″ N 49°35′12.5″ E ) from March 2019 to December 2020. A total of 150 cloacal swabs were collected from apparently healthy broilers in 15 different backyard chicken flocks (10 samples from each flock). Selected backyard flocks size ranged from 20 to 180 (median = 80) chickens per flock. Chickens were fed a balanced diet of protein, carbohydrates, vitamins, and minerals without antibiotic additives. Swabs were collected by random capture of birds and transported at 4 °C to the laboratory for bacteriological examination.

### 4.2. Bacterial Isolation

Swabs were cultured on BD™ Enterococcosel™ Agar (Heidelberg, Germany) and incubated at 37 °C for 24 h. Colonies exhibiting a black hallo were selected and subcultured on 5% sheep blood agar (Oxoid, UK) for purification. Purified isolates were identified based on colony morphology, Gram staining, catalase, and oxidase tests. Further, species-level identification was conducted biochemically using GP identification cards and the automated Vitek 2 compact system (BioMérieux, France).

### 4.3. DNA Extraction and 16S rRNA Gene Amplification and Sequencing

Biochemically identified isolates were cultured in brain heart infusion broth (Oxoid, UK) at 37 °C for 48 h. Cells were harvested by centrifugation, and the bacterial DNA was extracted and purified using the QIAamp DNA mini-kit (Qiagen SA, Courtaboeuf, France) according to the manufacturer’s instructions. The *16S rRNA* gene was amplified and sequenced according to Weisburg et al. [109] and further analyzed using the National Center for Biological Information (NCBI) Basic Local Alignment Search Tool (https://blast.ncbi.nlm.nih.gov/Blast.cgi, accessed on 15 December 2021). 

### 4.4. Antimicrobial Sensitivity Test

Ten antimicrobials, i.e., vancomycin (VAN, ≥4 μg/mL), erythromycin (ERY, ≥0.5 μg/mL), ampicillin (AMP, ≥8 μg/mL), nitrofurantoin (NIT, ≥32 μg/mL), tetracycline (TET, ≥4 μg/mL), linezolid (LZD, ≥2 μg/mL), chloramphenicol (CHL, ≥8 μg/mL), ciprofloxacin (CIP, ≥1 μg/mL), rifampicin (RIF, ≥1 μg/mL), and fosfomycin (FOS, ≥64 μg/mL) (Merck KGaA, Darmstadt, Germany), were selected for enterococcus antimicrobial sensitivity testing. Each antibiotic’s minimum inhibitory concentration (MIC) was established using the CLSI 202 criteria and recommendations [110]. Multidrug resistance (MDR) was considered when isolates were resistant to three or more different antimicrobial classes [111], and the MAR index was calculated using the methodology outlined by Krumperman et al. [112]. Calculation of MIC50 and MIC90 (equivalent to the median MIC value) was performed according to Schwarz et al. [113].

### 4.5. Detection of Antimicrobial-Resistance Genes

Antimicrobial-resistance genes associated with vancomycin (*vanA*, *vanB*), erythromycin (*ermA*, *ermB*), tetracycline (*tetA*, *tetB*, *tetM*, *tetL*), chloramphenicol (*cat*), linezolid (*optrA*), and ampicillin(*pbp5*) were determined by PCR [11,114,115,116,117,118,119,120]. Primers and PCR conditions are presented in Supplementary Table S2.

### 4.6. Phenotypic Detection of Virulence Factors

#### 4.6.1. Quantitative Biofilm Assay

Antimicrobial Biofilm formation was assessed according to the methods described by Stepanović et al. [121]. Enterococci from an overnight culture were cultivated in trypticase soy broth (TSB) supplemented with 1% glucose and incubated for 24 h at 37 °C. The culture density was adjusted to an approximate 0.5 McFarland standard. Each culture was diluted in sterile TSB (1:100), and 200 μL from each was transferred to three wells of sterile 96 well polystyrene microtiter plates (Sigma-Aldrich, St. Louis, MO USA). A sterile TSB was used as a negative control, and *E. faecalis* (ATCC 29212) was used as a positive control. The plates were incubated at 37 °C for 48 h, washed with sterile phosphate-buffered solution, air-dried, and stained with 2% crystal violet for 30 min. Subsequently, the wells were gently washed with sterile deionized water and air-dried. The dye bound to the adherent cells was re-solubilized with absolute ethanol (150) µL. Each well’s optical density (OD) was measured at 570 nm in a plate reader (BioTek-800 ST, St. Louis, MO USA). The experiment was performed in triplicate on three different days. Each Enterococcus isolate was classified as a negative, weak, moderate, or strong biofilm producer following the criteria described by Stepanović et al. [121].

#### 4.6.2. Gelatinase Activity

Gelatinase activity was assessed by inoculating pure culture on agar plates containing 3% gelatin [122]. After 48 h incubation, plates were flooded with a saturated ammonium sulfate solution. A transparent halo zone surrounding the colonies was considered positive for gelatinase.

#### 4.6.3. Cytolysin Activity

For screening hemolysin production, *Enterococcus* isolates were streaked on Columbia agar supplemented with 5% horse blood and incubated at 37 °C for 24 h. A clear (ß-hemolysis) or green (α hemolysis) zone around the colonies was defined as positive, whereas the γ-hemolysis was defined as negative activity [123].

### 4.7. Molecular Detection of Virulence Factor Genes

The virulence factor genes, including *gelE* (gelatinase), *agg* (aggregation substance), and *cylA* (activator of cytolysin), were screened by PCR, according to [124,125]. Primers and PCR conditions are tabulated in Supplementary Table S2.

### 4.8. Multi-Locus Sequence Typing

MLST for *E. faecalis* and *E. faecium* was performed by sequencing seven housekeeping genes described by Ruiz-Garbajosa et al. and Homan et al. [22,23]. Different sequences were assigned allele numbers, and different allelic profiles were assigned STs based on the MLST database (http://www.mlst.net/databases/, accessed: 15 December 2021).

### 4.9. PCR for repA Genes (Plasmid Families)

All MDR isolates were screened for rep-like sequences by PCR according to Jensen et al. [37] with primers and PCR conditions listed in Supplementary Table S2.

### 4.10. Statistical Analysis

The Fisher’s exact test or Chi-square test and Spearman’s rank correlation test were used for statistical analyses of the data (Prism 8 GraphPad Software, San Diego, CA, USA). Alignment and phylogenetic reconstructions were performed using the function “build” of ETE3 v3.1.1 [126] as implemented on the GenomeNet (https://www.genome.jp/tools/ete/, accessed: 15 December 2021). The tree was constructed using the Interactive Tree of Life (iTOL) v6.4.3 tool (https://itol.embl.de/, accessed: 15 December 2021) [127].

## 5. Conclusions

This study investigated virulence genes, antibiotic resistance, multi-locus sequence types, plasmid-associated genes, and the biofilm production of enterococci from healthy backyard chickens in Saudi Arabia to highlight the role of backyard chickens as a potential reservoir for MDR and virulent enterococci. Molecular analyses revealed the presence of nosocomial-associated CC17 and a variety of mobile genetic elements among the enterococci from backyard chickens, suggesting the possibility of their dissemination in backyard farms and their environments. 

High resistance to different antimicrobial classes was identified, suggesting the over-use of antimicrobials in backyard chicken farms. The emergence of MDR and virulent enterococci in backyard chicken farms is a public health concern. Thus, regular surveillance for the occurrence of MDR and virulent enterococci in backyard poultry and its environment is recommended to prevent its spread and to minimize environmental contamination.

Furthermore, proactive antimicrobial agent control measures should be developed to limit the spread of MDR enterococci.

## Figures and Tables

**Table 1:** Frequency of antimicrobial resistance and MIC values to 10 different antimicrobials for *E. faecalis*, *E. faecium*, *E. hirae* and *E. avium* isolated from backyard chickens.

Antimicrobial Agents	*E. faecalis* (*n* = 45)			*E. faecium* (*n* = 30)			*E. hirae* (*n* = 12)			*E. avium* (*n* = 3)		
	%R	MIC50	MIC90	%R	MIC50	MIC90	%R	MIC50	MIC90	%R	MIC50	MIC90
Ampicillin	15.6	2	32	33.3	4	64	66.7	16	32	66.7	32	32
Rifampin	24.4	1	16	10	1	1	0	0.5	1	0	1	1
Ciprofloxacin	13.3	1	8	23.3	1	16	33.3	1	8	66.7	4	8
Fosfomycin	17.8	32	256	13.3	32	256	8.3	32	256	0	32	64
Nitrofurantoin	22.2	16	256	16.7	16	256	8.3	16	32	0	32	32
Linezolid	0	1	2	0	1	2	0	1	2	0	1	2
Vancomycin	3.3	2	4	3.3	2	4	0	2	4	0	2	4
Chloramphenicol	8.9	4	8	6.7	4	8	0	4	8	0	4	8
Tetracycline	62.2	32	64	50	4	32	41.7	4	32	66.7	16	32
Erythromycin	31.1	0.5	32	16.7	16	256	33.3	0.5	16	0	0.5	0.5

**Table 2:** Antimicrobial-resistance profile of *Enterococcus* isolates (*n* = 90).

Resistance Profile	Number of Isolates	%Isolates
	16	17.8
FOS	3	3.3
TCY	4	4.4
ERY	2	2.2
CIP FOS	1	1.1
TCY RIF	2	2.2
TCY FOS	2	2.2
TCY CIP	3	3.3
NIT TCY	5	5.6
AMP CIP	5	5.6
AMP CHL	1	1.1
AMP TCY	4	4.4
AMP NIT	2	2.2
ERY RIF	2	2.2
ERY CHL	2	2.2
ERY TCY	3	3.3
ERY NIT	2	2.2
VAN TCY	1	1.1
TCY CHL RIF	1	1.1
NIT TCY RIF	2	2.2
AMP TCY FOS	3	3.3
AMP TCY CIP	3	3.3
ERY TCY RIF	2	2.2
ERY TCY FOS	1	1.1
ERY TCY CIP	3	3.3
ERY NIT RIF	2	2.2
ERY NIT TCY	1	1.1
ERY PEN TCY	6	6.7
AMP TCY CHL CIP	1	1.1
AMP NIT CIP FOS	1	1.1
ERY CHL CIP FOS	1	1.1
VAN NIT TCY RIF	1	1.1
VAN ERY TCY RIF	1	1.1
AMP TCY CIP FOS RIF	1	1.1

FOS = Fosfomycin; TCY = tetracycline; ERY = erythromycin; CIP = ciprofloxacin; VAN = vancomycin; PEN = penicillin; NIT = nitrofurantoin; LZD = linezolid; CHL = chloramphenicol; RIF = rifampicin.

**Table 3:** Distribution of antimicrobial-resistance genes among the isolated enterococci.

	*E. faecalis* (N = 45)	*E. faecium* (N = 30)	*E. hirae* (N = 12)	*E. avium* (N = 3)	Total Enterococci (N = 90)
*vanA*	1	0	0	0	1
*vanB*	0	0	0	0	0
*ermA*	5	4	2	0	11
*ermB*	13	9	2	0	24
*pbp5*	7	8	6	2	23
*tetA*	1	0	0	0	1
*tetB*	0	0	0	1	1
*tetM*	26	8	3	2	39
*tetL*	15	12	5	2	34
*optrA*	0	0	0	0	0
*Cat*	3	1	0	0	4

**Table 4:** Distribution of mean MAR index and virulence genes among different biofilm categories.

Biofilm Category	Mean Biofilm OD	Mean MAR Index	MDR	Gelatinase	Cytolysin	Agg	gelE
Non-biofilm producers	0.29	0.017	0	0	0	0	0
Weak biofilm producers	0.47	0.13	0	3	0	2	2
Medium biofilm producers	0.9	0.22	8	3	3	8	7
Strong biofilm producers	1.65	0.3	22	9	9	23	16

**Table 5:** Multi-locus sequence types and allele numbers for 45 *E. faecalis* strains isolated from backyard chickens.

ST	NO	MLST Allelic Profile							Phenotypic Activities			
		*gdh*	*gyd*	*pstS*	*gki*	*aroE*	*xpt*	*yqiL*	Biofilm	MDR	Gelatinase	Cytolysin
16	10	5	1	1	3	7	7	6	10	6	2	3
21	3	1	7	9	1	1	1	1	1	0	0	0
32	1	8	7	9	5	4	4	1	1	0	0	0
41	5	1	7	11	21	1	4	1	3	0	0	1
81	1	27	2	16	28	26	2	1	1	1	1	0
176	2	15	7	3	37	39	15	11	2	0	1	0
177	1	15	2	37	37	39	15	11	0	0	0	0
179	6	5	1	1	3	7	1	6	5	2	3	2
302	8	5	1	1	3	7	7	60	8	4	5	2
480	5	1	1	22	22	7	17	6	5	2	0	1
752	3	27	2	16	28	26	83	1	3	0	0	0

**Table 6:** Multi-locus sequence types and allele numbers for 30 *E. faecium* strains isolated from backyard chickens.

ST	NO	MLST Allelic Profile							Phenotypic Activities			
		*atpA*	*ddl*	*gdh*	*purK*	*gyd*	*pstS*	*adk*	Biofilm	MDR	Gelatinase	Cytolysin
12	1	5	2	6	6	1	7	1	0	0	0	0
360	2	5	2	6	6	1	1	1	0	0	0	0
16	3	1	2	1	1	1	1	1	2	1	0	0
194	8	1	1	1	1	1	1	1	8	4	2	1
157	5	7	1	1	1	5	1	1	5	2	0	1
9	4	4	5	1	3	1	1	1	4	0	0	0
18	2	15	1	1	1	1	1	1	1	0	1	0
82	5	1	36	1	1	1	1	1	4	1	0	1

**Table 7:** Rep-families, phenotypic and genotypic resistance profile detected among MDR enterococci from backyard chickens.

*Enterococcus* spp.	Phenotypic Profile	AMR Genes	Virulence Genes	Gelatinase	Cytolysin	Rep-Family	Plasmid
*E. faecalis*	AMP, ERY, TET	*pbp5*, *tetM*, *tetL*	*gelE*, *agg*	−	−	9	pCF10
*E. faecalis*	AMP, TET, FOS	*pbp5*, *tetM*	*gelE*, *agg*, *cylA*	−	−		
*E. faecalis*	ERY, NIT, TET	*ermA*, *ermB*, *tetM*	*gelE*, *agg*	+	−	9	pCF10
*E. faecalis*	ERY, CHL, CIP, FOS	*ermB*	*gelE*, *agg*, *cylA*	+	−		
*E. faecalis*	VAN, ERY, TET, RIF	*vanA*, *ermB*, *tetM*, *tetL*	*gelE*, *agg*	+	−	9, 1	pIP501, pCF10
*E. faecalis*	ERY, NIT, RIF	*ermA*	*gelE*, *agg*, *cylA*	−	+		
*E. faecalis*	ERY, NIT, RIF	*ermB*	*gelE*, *agg*	−	−		
*E. faecalis*	AMP, TET, CIP, RIF, FOS	*pbp5*	*gelE*, *agg*, *cylA*	−	+	6	pS86
*E. faecalis*	TET, CHL, RIF	*tetM*, *cat*		−	−		
*E. faecalis*	AMP, TET, FOS	*pbp5*, *tetM*	*gelE*, *agg*	+	−		
*E. faecalis*	AMP, TET, FOS	*pbp5*, *tetM*, *tetL*	*gelE*, *agg*, *cylA*	+	+	9	pCF10
*E. faecalis*	ERY, TET, RIF	*ermB*, *tetM*	*gelE*, *agg*	+	−	9	pCF10
*E. faecalis*	NIT, TET, RIF	*tetM*	*gelE*, *agg*	+	−	6	pS86
*E. faecalis*	ERY, TET, RIF	*ermB*, *tetL*		−	−	9	pCF10
*E. faecalis*	NIT, TET, RIF		*gelE*, *agg*, *cylA*	−	−		
*E. faecium*	AMP, TET, CIP	*pbp5*, *tetM*, *tetL*	*agg*	−	−	2	pRE25
*E. faecium*	VAN, NIT, TET, RIF	*vanA*, *tetM*, *tetL*	*agg*	−	−	2, 1	pIP501, pRE25
*E. faecium*	AMP, TET, CHL, CIP	*pbp5*, *tetM*, *tetL*, *cat*	*geIE*, *agg*	+	−	2	pRE25
*E. faecium*	ERY, AMP, TET	*ermA*, *ermB*, *tetL*	*cylA*	−	−		
*E. faecium*	ERY, TET, CIP	*tetL*	*gelE*, *agg*, *cylA*	−	+	2, 6	pRE25, pS86
*E. faecium*	ERY, TET, CIP	*ermB*	*agg*	−	−		
*E. faecium*	ERY, TET, CIP	*ermA*, *ermB*	*geIE*, *agg*	+	−	6	pS86
*E. faecium*	ERY, TET, FOS	*ermA*, *ermB*, *tetM*, *tetL*	*agg*, *cylA*	−	+	2	pRE25
*E. hirae*	ERY, AMP, TET	*tetL*	*agg*	−	−		
*E. hirae*	ERY, AMP, TET	*ermA*, *ermB*, *pbp5*, *tetM*, *tetL*		−	−	5	pN315
*E. hirae*	ERY, AMP, TET	*ermA*, *ermB*, *pbp5*, *tetL*	*gelE*, *agg*, *cylA*	−	+	5,3	pN315, pAW63
*E. hirae*	AMP, NIT, CIP, FOS	*pbp5*	*geIE*, *agg*	−	−	6	pS86
*E. hirae*	ERY, AMP, TET	*ermA*, *ermB*, *pbp5*, *tetM*, *tetL*	*agg*	−	+	5, 11	pN315, pEF1071
*E. avium*	AMP, TET, CIP	*pbp5*, *tetB*, *tetM*, *tetL*		−	−	6	pS86
*E. avium*	AMP, TET, CIP	*pbp5*, *tetM*, *tetL*	*geIE*	−	−	6, 9	pS86, pCF10

## Data Availability

The data presented in this study are available on request from the corresponding author.

## Supplementary Materials

The following are available online at https://www.mdpi.com/article/10.3390/antibiotics11030380/s1, Figure S1: Description and categories of the collected variables. Table S1: Description and categories of the collected variables. Table S2: Description and categories of the collected variables.
