# Supplementary material for: Antimicrobial Resistance, Biofilm Formation, and Virulence Genes in Enterococcus Species from Small Backyard Chicken Flocks
Source: Antibiotics (Basel). 2022 Mar 13;11(3):380. doi: 10.3390/antibiotics11030380 (PMC8944505; doi:10.3390/antibiotics11030380)
Supplement: Supplementary file 1 [file antibiotics-11-00380-s001.zip › antibiotics-1559112-supplementary.pdf]

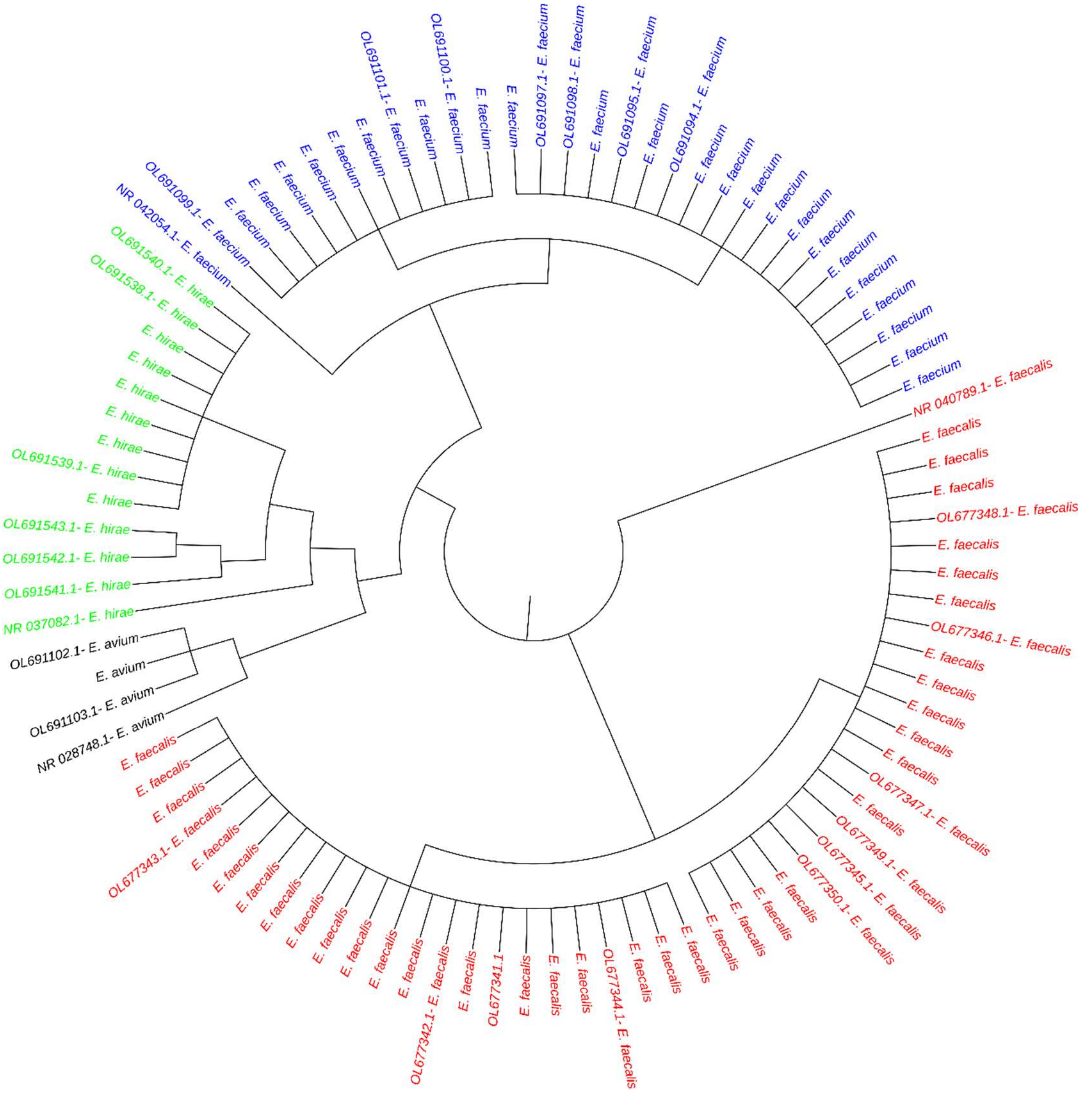

Figure S1. Phylogenetic tree based on 16S rRNA sequences of *E. faecalis*, *E. faecium*, *E. hirae* and *E. avium* isolates recovered from backyard chickens.

Table S1. MIC values and the resistance levels to ten different antimicrobials for enterococci isolates (n=90).

Antibiotic name	Breakpoints	spp	Number	%R	%R 95%C.I.	MIC50	MIC90	MIC Range	0.064	0.125	0.25	0.5	1	2	4	8	16	32	64	128	256	>256
Ampicillin	S<=8 R>=16	*E. faecalis*	45	15.6	7.0-30.1	2	32	0.5 - 64				4	4	16	11	3	0	6	1			
		E. faecium	30	33.3	17.9-52.8	4	64	1 - 64					1	5	12	2	2	3	5			
		E. hirae	12	66.7	35.5-88.7	16	32	1 - 32					1	2		1	3	5				
		E. avium	3	66.7	12.5-98.2	32	32	2 - 32						1				2				
Rifampin	S<=1 R>=4	*E. faecalis*	45	24.4	13.4-39.8	1	16	0.125 - 32		2	1	10	21		1	3	6	1				
		E. faecium	30	10	2.6-27.7	1	1	0.5 - 16				6	21			2	1					
		E. hirae	12	0	0.0-30.1	0.5	1	0.5 - 1				6	6									
		E. avium	3	0	0.0-69.0	1	1	1 - 1					3									
Ciprofloxacin	S<=1 R>=4	*E. faecalis*	45	13.3	5.5-27.4	1	8	0.125 - 16		5	6	9	19		1	4	1					
		E. faecium	30	23.3	10.6-42.7	1	16	0.125 - 16		3	4	4	12		2	1	4					
		E. hirae	12	33.3	11.3-64.5	1	8	0.125 - 8		3		1	4		2	2						
		E. avium	3	66.7	12.5-98.2	4	8	0.5 - 8				1			1	1						
Fosfomycin	S<=64 R>=256	*E. faecalis*	45	17.8	8.5-32.6	32	256	2 - 512						1		4	6	18	8		5	3
		E. faecium	30	13.3	4.3-31.6	32	256	16 - 512									7	12	7		1	3
		E. hirae	12	8.3	0.4-40.2	32	256	8 - 256								1	2	3	5		1	
		E. avium	3	0	0.0-69.0	32	64	32 - 64										2	1			
Nitrofurantoin	S<=32 R>=128	*E. faecalis*	45	22.2	11.7-37.5	16	256	4 - 256							4	6	18	7		4	6	
		E. faecium	30	16.7	6.3-35.5	16	256	4 - 256		8	1	11				2	7	1				
		E. hirae	12	8.3	0.4-40.2	16	32	4 - 128							3		5	3		1		
		E. avium	3	0	0.0-69.0	32	32	16 - 32									1	2				
Linezolid	S<=2 R>=8	*E. faecalis*	45	0	0.0-9.8	1	2	0.125 - 2		1	1	7	29	7								
		E. faecium	30	0	0.0-14.1	1	2	0.25 - 2			2	3	20	5								
		E. hirae	12	0	0.0-30.1	1	2	0.25 - 2			1	2	5	4								
		E. avium	3	0	0.0-69.0	1	2	1 - 2					2	1								
Vancomycin	S<=4 R>=32	*E. faecalis*	45	4.4	0.8-16.3	2	4	1 - 32					10	29	4			2				
		E. faecium	30	3.3	0.2-19.0	2	4	1 - 32					3	18	8			1				
		E. hirae	12	0	0.0-30.1	2	4	1 - 4					1	6	5							
		E. avium	3	0	0.0-69.0	2	4	1 - 4					1	1	1							
Chloramphenicol	S<=8 R>=32	*E. faecalis*	45	8.9	2.9-22.1	4	8	1 - 64					1	12	20	8			4			
		E. faecium	30	6.7	1.2-23.6	4	8	2 - 64						7	16	5			2			
		E. hirae	12	0	0.0-30.1	4	8	2 - 8						2	5	5						
		E. avium	3	0	0.0-69.0	4	8	2 - 8						1	1	1						
Tetracycline	S<=4 R>=16	*E. faecalis*	45	62.2	46.5-75.8	32	64	0.5 - 64				1	2	10	4		5	18	5			
		E. faecium	30	50	31.7-68.3	4	32	1 - 64					2	8	5		5	8	2			
		E. hirae	12	41.7	16.5-71.4	4	32	1 - 32					2	3	2		2	3				
		E. avium	3	66.7	12.5-98.2	16	32	2 - 32						1			1	1				
Erythromycin	S<=.5 R>=8	*E. faecalis*	45	31.1	18.6-46.8	0.5	32	0.06 - 64	3	15	4	9					8	4	2			
		E. faecium	30	16.7	6.3-35.5	16	256	4 - 256							1	2	17	5		1	4	
		E. hirae	12	33.3	11.3-64.5	0.5	16	0.125 - 16		1	4	3				2	2					
		E. avium	3	0	0.0-69.0	0.5	0.5	0.25 - 0.5			1	2										

Table S2. Primers and PCR condition for detection of virulence and antimicrobial resistance genes and Rep-families

Genes	Oligonucleotide sequences (5′-3′)	Length (bp)	Annealing temperature	References
vanA	GCGCGGTCCACTTGTAGATA<br>TGAGCAACCCCCAAACAGTA	314	54	[114]
vanB	AGACATTCCGGTCGAGGAAC<br>GCTGTCAATTAGTGCGGGAA	220	54	[114]
Cat	GGATATGAAATTTATCCCTC<br>CAATCATCTACCCTATGAAT	486	50	[11]
ermA	GCGGTAAACCCCTCTGAG<br>GCCTGTCGGAATTGG	434	51	[115]
ermB	CATT TAACGACGAAACTGGC<br>GGAACATCTGTGGTATGGCG	425	51	[116]
tetM	GTGGACAAAGGTACAACGAG<br>CGGTAAAGTTCGTCACACAC	406	57	[117]
tetA	GCT ACA TCC TGC TTG CCTTC<br>CAT AGA TCG CCG TGA AGAGG	210	58	[117]
tetB	TTG GTTA GGG GCA AGT TTTG<br>GTA ATG GGC CAA TAA CACCG	659	58	[117]
tetL	ATAAATTGTTTCGGGTCGGTAAT<br>AACCAGCCAACTAATGACAATGAT	1,077	55	[118]
optrA	AGGTGGTCAGCGAACTAA<br>ATCAACTGTTCCCATTCA	1395	55	[119]
pbp5	AACAAAATGACAAACGGG<br>TATCCTTGGTTATCAGGG	779	54	[120]
gelE	TATGACAATGCTTTTTGGGAT<br>AGATGCACCCGAAATAATATA	312	56	[125]
cylA	ACTCGGGGATTGATAGGC<br>GCTGCTAAAGCTGCGCTT	688	56	[125]
Agg	CCAGTAATCAGTCCAGAAACAACC<br>TAGCTTTTTTCATTCTTGTGTTTGTT	406	54	[124]
rep1(pIP501)	TCGCTCAATCACTACCAAGC<br>CTTGAACGAGTAAAGCCCTT	624	56	[37]
rep2 (pRE25)	GAGAACCATCAAGGCGAAAT<br>ACCAGAATAAGCACTACGTACAATCT	630	56	
rep3 (pAW63)	CCTAATGTATATAATTTTGGTACATAT<br>ACATTTTCCTCAAAGAACAT	304	52	
rep4 (pMBB1)	ACTATGTCGTTGAGTCTAATGACT<br>AGCAAGATAGAATATTTACTTTTAAGTTT	304	52	
rep5 (pSAS)	ATGTGTAATAAATTAAAAGAGCA<br>ATTGTCTTGATTTATCTATCTTG	637	52	
rep6 (pS86)	ACGAATGAAAGATAAAGGAGTAG<br>TAAATTCTAGTTTGGCAATCTTAT	551	56	
rep7 (pUSA02)	CCAAAATAYTTYGTTTCTGG<br>CCAATCATGTAATGTTACAACC	227	56	
rep8 (pAM373)	TAGATACGACAAAAGAAGAATTACA<br>CCAATCATGTAATGTTACAACC	394	56	
rep9 (pCF10)	GCTCGATCARTTTTCAGAAG<br>CGCAAACATTTGTCWATTTCTT	210	56	
rep10 (pIM13)	TATAAAGGCTCTCAGAGGCT<br>CCAAATTCGAGTAAGAGGTA	382	56	
rep11(pEF1071)	TCTAGAATGCGTAAAAAGG<br>CCTTTGAAGATWGCRGTWAG	500	52	
rep12 (pBMB67)	GAGCCTATAACAGAGTACACA<br>CAAATATAGGCTTTGTAGTTC	470	52	
rep13 (pC194)	ATGATGCAATATATTAAGCA<br>TACCAGAATAYTTAGCCATTTC	402	52	
rep14(pRI)	GAAAGYTTRGATAGYTTTGC<br>RTTTTGRCTTTCTTSYTTCA	164	52	
rep15(pUSA03)	CAGTAGAAGAAAATTATAAAGAAC<br>GTTATGGCTGGTTTTAATAAA	327	52	
rep16 (pSAS)	CAGGAAAACACTTCGTTTAT<br>CTTCTATATCACTATCATTGTCATT	592	52	
rep17 (pRUM)	TACTAACTGTTGGTAATTCGTTAAAT<br>ATCAAGGACTCAACCGTAATT	604	52	
rep 18 (pEF418)	ACACCAGTCGAAATGAATTT<br>AGGAATATCAAGTAATTCATGAAAGT	462	56	
rep19 (pUB101)	GWGATCGCTTARAYTTATCTAT<br>YMTTGTTSTGGMAATTCTT	543	52	
